# Understanding Primary Care Physicians’ Comprehension of Eye Care Notes to Improve Diabetes Care Coordination

**DOI:** 10.21203/rs.3.rs-10146623/v1

**Published:** 2026-07-03

**Authors:** Lauren E. Wedekind, Fritz Gerald P. Kalaw, Helena Gali, Kristen Kulasa, Kendall Moore, Quinn Quackenbush, Sally L. Baxter, Christine Thorne

**Affiliations:** University of California San Diego; University of California San Diego; Vantage Eye Center; University of California San Diego; University of California San Diego; University of California San Diego; University of California San Diego; University of California San Diego

**Keywords:** ophthalmology, EHR, notes, diabetes, diabetic retinopathy

## Abstract

**Background:**

Diabetic retinopathy screening and management involve collaboration between primary care providers (PCPs), endocrinologists, and eye care providers, yet eye care documentation poses challenges to inter-specialty communication.

**Objective:**

To assess PCP and endocrinologist comprehension of ophthalmology notes, identify barriers to interpretation, and identify strategies for improvement.

**Methods:**

We conducted a cross-sectional online survey of 82 PCPs and endocrinologists across three academic tertiary health systems between November 2022 and December 2024. The survey collected demographic data, prior rotations in ophthalmology, and self-reported comprehension of components of eye care notes using 5-point Likert scales. 67 participants reviewed three sample eye care progress notes and identified whether a dilated fundus examination was performed and the presence of diabetic retinopathy for each note. Free-text responses were analyzed thematically.

**Results:**

Self-reported comprehension of eye care documentation was modest: 47% agreed and 6% strongly agreed that they generally understand eye care notes. Comprehension of sample notes similarly varied, with correct identification of dilation status ranging from 94–96% and diabetic retinopathy status from 37–82%. 29% of respondents who answered fewer than half of the questions correctly reported confidence in their general understanding of eye care notes. Major barriers to inter-specialty care coordination for patients with diabetes included inconsistent coding and formatting and use of ophthalmology-specific jargon and abbreviations.

**Conclusions:**

PCPs and endocrinologists demonstrated variable confidence in their comprehension of eye care documentation, with measurable gaps in interpreting key clinical elements of notes. More standardized and structured documentation may improve inter-specialty communication and coordination of care for patients with diabetes.

## Introduction

Diabetic retinopathy is the most common microvascular complication of diabetes mellitus and is a leading cause of preventable blindness in the United States (U.S.).^1^ According to the Office of Disease Prevention and Health Promotion, only 64.8% of U.S. adults aged 18 years and older with diagnosed diabetes had an eye exam within the past 12 months in 2019.^2^ Patients with diabetes require coordinated care between eye care providers (ophthalmologists and optometrists), primary care providers (PCPs – family medicine and internal medicine physicians), and endocrinologists. Even with shared electronic medical records, eye care documentation is often characterized by abbreviations and specialty-specific terminology, may limit effective communication across specialties.^3,4,5,6^ This study aimed to assess comprehension of ophthalmology notes among PCPs and endocrinologists and to identify barriers to interpretation.

## Methods

### Study Design

This quality improvement study was reviewed by the University of California San Diego (UCSD) IRB # 810632. The study protocol, survey tool, participant information sheet (consent), and recruitment flier were reviewed by the IRB and approved as an exempt study. The study adhered to the Principles of the Declaration of Helsinki.

### Survey Characteristics

An online survey was distributed to primary care physicians and endocrinologists at three academic health systems (University of California Davis, University of California Los Angeles, and UCSD) between November 2022 and December 2024 using the Qualtrics Experience Management (XM) survey platform. Distribution occurred both in-person and virtually through emails to faculty, QR code distribution at faculty/staff meetings (in person and remote), and through fliers with QR codes placed on-site at primary care and endocrinology clinics in physician work areas. Participation was voluntary, and informed consent was obtained from all survey respondents using the approved IRB consent at the start of the survey.

The survey collected demographic data, prior ophthalmology exposure, and self-reported comprehension of sample eye care notes using 5-point Likert scale. Participants reviewed 3 sample eye care progress notes (Figure S1) and were asked to identify for each: (1) whether a dilated fundus examination was performed and (2) diabetic retinopathy status. A voluntary free-text section invited respondents to share additional comments regarding diabetes care coordination with eye care providers and comprehension of eye care notes. Participants were included if they completed 70% or more of all questions.

### Analysis

Descriptive statistics were calculated using Microsoft Excel version 16.58 (Microsoft Corporation). Continuous variables are reported as means (standard deviations, SD) and categorical and binary variables were described using counts (percentages). When skipped, yes/no questions were considered to have an answer of no. Free-text comments were reviewed using thematic analysis to identify themes of suggestions on how eye care providers could improve primary care physicians’ and endocrinologists’ comprehension of eye care notes.

## Results

82 physicians completed 70% or more of survey questions; subgroup analyses were performed for participants who answered questions about perceived comprehension of eye care notes (n = 72) and completed the task of reviewing sample eye care notes (n = 67). 54% of all 82 participants identified as female, 51% as White, 23% as Asian, and 22% preferring not to disclose (Table 1). The mean age was 47 years (SD 10.1). The specialty with the greatest number of respondents was internal medicine-primary care (39%). Participants had a mean practice duration of 12.7 years (SD 7.5). 39% of respondents had completed ophthalmology rotations during medical school, with a mean duration of 3.5 weeks. 23% of respondents had completed ophthalmology rotations during residency, with a mean duration of 2 weeks.

When asked whether they generally read their patients’ eye care notes, 29% of respondents answered “always,” 65% answered “sometimes,” 2% answered “never”, and 4% did not answer.

A subset of 72 respondents completed specific questions on perceptions of their own comprehension of eye care notes, which varied across notes and question types (Table 2).

While 76% or strongly agreed that they understood the assessment and plan, fewer reported confidence in their understanding of eye examination findings (39%) and overall note content (53%). Only 6% of respondents strongly agreed that they generally understand eye care notes. A substantial proportion selected neutral responses (16–33%). Respondents most commonly used online search engines when they did not understand parts of eye care progress notes (68%). Some also consulted the referred eye care provider (26%) or other ophthalmology or optometry colleagues (15%).

67 respondents evaluated sample eye care notes (Table 2).

**Figure 1:**
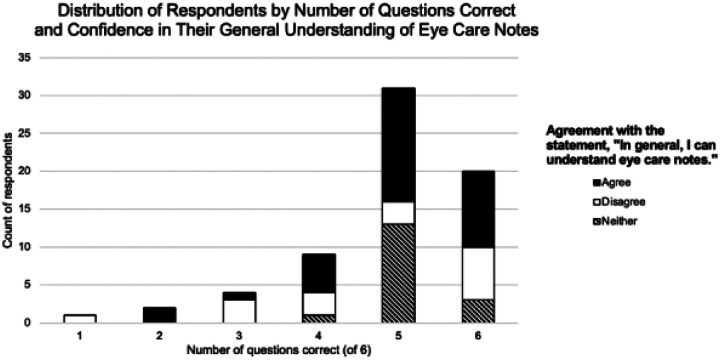
Relationship between eye care note comprehension and self-reported ability to understand eye care notes (n = 67). Respondents are grouped by the number of eye care note comprehension questions they answered correctly (x-axis; maximum score of 6). The height of each stacked bar represents the number of respondents and differently colored bar segments indicate respondents’ level of agreement with the statement, “In general, I can understand eye care notes.”

Nearly all (94–96%) accurately determined dilation status. By contrast, accuracy for diabetic retinopathy status ranged from 37% to 82%, with up to 58% uncertain on the correctness of their answers. Overall, 77% of respondents answered 5 to 6 questions correctly (Fig. 1).

Most respondents did not respond to questions about their confidence in each of their individual responses; thus, we assessed the distribution of respondents based on the number of questions answered correctly and their confidence in their general understanding of eye care notes. Those who answered 5 of 6 questions correctly had similar rates of reporting confidence in their answers (“agree” category) to those who answered all questions correctly (48% vs. 50%), as well as lower rates of saying they were not confident in their answers (10% vs. 35%). A greater proportion of those with 5 of 6 correct answers felt ambivalent about answers (42% vs. 15%) than those who answered all questions correctly. By contrast, 29% of respondents who answered 3 or more questions incorrectly reported being confident in their answers.

Free-text comments from respondents (Table 3) highlighted recurring challenges in interpreting eye care providers’ notes and provided suggestions to improve diabetes care coordination.

Key barriers included inconsistent linkage of diagnoses and medications in the electronic medical record, frequent use of abbreviations and ophthalmology-specific jargon, and variable note formatting. Suggested improvements included linking medications to diagnoses, maintaining updated problem lists, employing automated expansion of abbreviations, and adopting standardized note templates that clearly document diabetic retinopathy screening status.

## Discussion

This study demonstrates that primary care physicians and endocrinologists experience barriers to understanding eye care notes, with difficulty interpreting diabetic retinopathy status and uncertainty about their level of comprehension. Although preclinical teaching in ophthalmology has been universal among Association of University Professors in Ophthalmology (AUPO)-affiliated medical schools, only 18% (20 of 113) require clinical exposure in ophthalmology.^7^ Bowers and colleagues^8^ reported that medical students who participated in one-on-one sessions with ophthalmology residents and didactic sessions with a course director reported they had a “good” level of exposure to ophthalmology after one week.^8^ 38% of our survey respondents participated in ophthalmology rotations during medical school, and 26% did so during residency. While these figures are greater than nationwide estimates, respondents reported difficulty in understanding ophthalmology notes. This highlights a gap in inter-specialty communication that may affect the coordination of care for patients with diabetes.

Abbreviations and ophthalmology-specific terminology were cited as barriers to inter-specialty care coordination. These conventions enable efficient documentation among eye care providers. Prior work has shown that ophthalmologists, with very limited time in patient encounters, spend brief amounts of time on documentation.^9^ Abbreviations carry a cost: especially when interpreted by providers in different specialties, non-standard abbreviations have been shown to contribute to communication errors.^10^ In our survey, the vast majority of respondents reported reviewing their patients’ eye care notes. One key finding in the current study was that only 6% of respondents had full confidence (strongly agree) in their understanding of eye care progress notes in general, as well as their understanding of eye examination findings. This was reflected when they were asked to interpret sample eye exam notes, with high variability of survey responses correctly identifying diabetic retinopathy status based upon note data. Standardized documentation practices, including structured templates and reduced use of ophthalmology-specific abbreviations, may improve interpretability without increasing documentation burden.

The use of online search engines was the most common approach for referring physicians to help understand unfamiliar abbreviations used by eye care providers. This is consistent with prior studies regarding information retrieval in medicine more broadly.^11^ Electronic health records support templated documentation and the ability to automatically convert specified acronyms to full terms.^12,13,14^ Large language models (LLMs) could potentially aid in “translating” specialized terminology into more accessible language for non-eye care providers.^15^

This study has several limitations, including a modest sample size, potential response bias, and restriction to academic medical centers in California, which may limit generalizability. In addition, respondents evaluated a limited number of sample notes, which may not reflect the full range of documentation formats. Despite these limitations, this study provides quantitative and qualitative evidence of a communication gap in ophthalmology documentation and identifies strategies to improve interdisciplinary care for patients with diabetes.

## Conclusion

PCPs and endocrinologists demonstrate variable comprehension of ophthalmology notes, with gaps in interpreting key clinical elements. More standardized and structured documentation, alongside emerging technologies, may improve the coordination of care across specialties for patients with diabetes.

## Supplementary Material

Supplementary Files

This is a list of supplementary files associated with this preprint. Click to download.

- WedekindLESciRepSuppInfo.docx

## Figures and Tables

**Table 1:** Summary of survey responses from primary care physicians and endocrinologists regarding comprehension of eye care notes (N = 82).

Demographic characteristics	N = 82	%
**Age** (mean in years [SD])	47 (10.1)*	
**Sex**		
Female	44	53.7%
Male	29	35.4%
Prefer not to disclose	9	11.0%
**Race**		
American Indian/Alaska Native	1	1.2%
Asian	19	23.2%
More than one	2	2.4%
White	42	51.2%
Prefer not to disclose	18	22.0%
**Clinical characteristics**		
**Specialty/Subspecialty**		
Family Medicine	30	36.6%
Internal Medicine – Primary Care	32	39.0%
Internal Medicine – Endocrinology	14	17.1%
Prefer not to disclose	6	7.3%
**Duration of practice (mean in years [SD])**	12.7 (7.5)**	
**Ophthalmology training and notes**		
**Ophthalmology rotation in medical school**		
No	50	61.0%
Yes (mean duration [weeks])	32 (3.5)	39.0%
**Ophthalmology rotation in residency**		
No	63	76.8%
Yes (mean duration [weeks])	19 (2.0)	23.2%
**Reads eye care progress notes**		
Always	24	29.3%
Sometimes	53	64.6%
Never	2	2.4%
Did not answer	3	3.7%

* one respondent did not disclose their age.

** one respondent was an endocrinology fellow.

SD – standard deviation

**Table 2:** Survey respondents regarding perceived comprehension of eye care notes (n = 72).

Agree	General comprehension of eye care notes	Understanding eye exam findings from the note	Understanding of the assessment and plan
	34 (47.2%)	28 (38.9%)	55 (76.4%)
Disagree	20 (27.8%)	20 (27.8%)	5 (6.9%)
Neither agree nor disagree	18 (25.0%)	24 (33.3%)	12 (16.7%)

**Table 2:** Comprehension of 3 sample eye care notes by question type (dilation and diabetic retinopathy, DR, status) (n = 67).

	Note 1		Note 2		Note 3	
	Dilation	DR	Dilation	DR	Dilation	DR
Correct	63 (94.0%)	53 (79.1%)	64 (95.5%)	55 (82.1%)	63 (94.0%)	25 (37.3%)
Incorrect	0 (0.0%)	5 (7.6%)	1 (1.5%)	4 (6.0%)	1 (1.5%)	1 (1.5%)
Unsure or blank	4 (6.0%)	9 (13.4%)	2 (3.0%)	8 (11.9%)	3 (4.5%)	39 (58.2%)

**Table 3.** Free-text comments from respondents with suggestions on how to improve primary care providers’ and endocrinologists’ comprehension of eye care notes, grouped by theme.

Theme	Example comment
**Charting**	“Would love if eye drops/prescriptions had the proper diagnosis associated, and notes clearly indicated how long patients are supposed to use those drops. Patients often ask primary care for refills as they can’t get a response from ophtho, but it’s not always clear to us.”
	“Eye specialists should update problem lists in shared EMRs.”
**Abbreviations**	“Templates, notes, and dictionaries should change abbreviations automatically to full words make this obsolete. E.g., could type ‘NPDR’ and it would type out nonproliferative diabetic retinopathy… This has worked well to eliminate unsafe abbreviations such as QD and QID.”
	“Extreme jargon in ophthalmology notes. The computer can translate… by dictionary replacement, if set up to do so.”
**Formatting**	“I would greatly appreciate if eye care providers could always include in their notes the date of patient’s last diabetic retinopathy screening and the result. It is confusing when a patient has multiple follow-ups for other eye conditions (e.g., cataract surgery), and no mention is made of DR, even though they have had multiple eye appointments.”
	“Sometimes notes are scanned handwritten notes, which adds to the challenge with interpretation.”

## Data Availability

All data in the current study are available from the corresponding author upon request.
